# Angiotensin-Converting Enzyme 2: The First Decade

**DOI:** 10.1155/2012/307315

**Published:** 2011-11-10

**Authors:** Nicola E. Clarke, Anthony J. Turner

**Affiliations:** Institute of Molecular and Cellular Biology, Faculty of Biological Sciences, University of Leeds, Leeds LS2 9JT, UK

## Abstract

The renin-angiotensin system (RAS) is a critical regulator of hypertension, primarily through the actions of the vasoactive peptide Ang II, which is generated by the action of angiotensin-converting enzyme (ACE) mediating an increase in blood pressure. The discovery of ACE2, which primarily metabolises Ang II into the vasodilatory Ang-(1-7), has added a new dimension to the traditional RAS. As a result there has been huge interest in ACE2 over the past decade as a potential therapeutic for lowering blood pressure, especially elevation resulting from excess Ang II. Studies focusing on ACE2 have helped to reveal other actions of Ang-(1-7), outside vasodilation, such as antifibrotic and antiproliferative effects. Moreover, investigations focusing on ACE2 have revealed a variety of roles not just catalytic but also as a viral receptor and amino acid transporter. This paper focuses on what is known about ACE2 and its biological roles, paying particular attention to the regulation of ACE2 expression. In light of the entrance of human recombinant ACE2 into clinical trials, we discuss the potential use of ACE2 as a therapeutic and highlight some pertinent questions that still remain unanswered about ACE2.

## 1. Introduction

When angiotensin-converting enzyme-2 (ACE2) was serendipitously discovered ten years ago, neither of the two groups at the centre of its discovery [1, 2] could have guessed at the disproportionate number of distinct roles it plays in biology, from cardiovascular regulation to viral infection. As so often happens in modern biological research two independent approaches converged on the same discovery, to give us ACE2 or angiotensin-converting enzyme homologue (ACEH), back in 2000. Over the past ten years our knowledge of this protein's role in the body has increased exponentially, resulting in recombinant ACE2 protein entering clinical trials back in 2009. This paper will focus on what we currently know about ACE2 and its regulation, highlighting some of the gaps and discrepancies that still remain in our knowledge. 

## 2. Biochemistry and Cell Biology of ACE2: Comparisons and Distinctions from ACE

**Figure 1:**
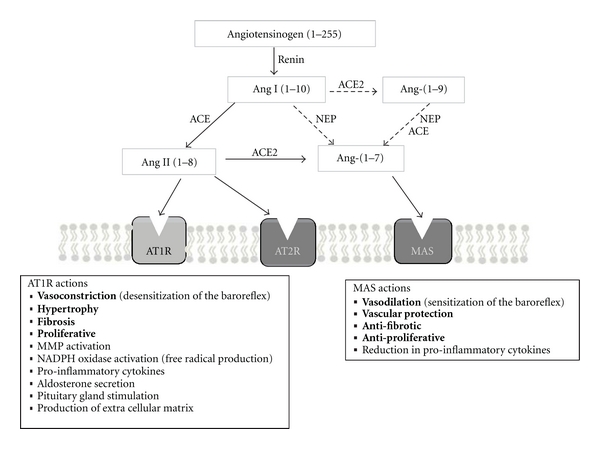
Schematic representation of the renin-angiotensin system (RAS). ACE: angiotensin-converting enzyme; ACE2: angiotensin-converting enzyme 2; NEP: neprilysin; AT1R: Ang II type 1 receptor. Angiotensinogen is cleaved by renin in the circulation to generate Ang I. Ang I is cleaved to yield Ang II by ACE, Ang-(1-7) by NEP, or Ang (1-9) by ACE2; this reaction is much less favourable than the production of Ang-(1-7) from Ang II. Ang-(1-9) is then cleaved by either NEP or ACE to yield Ang-(1-7) in a minor pathway. Ang II exerts its main actions by binding to the AT1R. Ang II can also be further cleaved by ACE2, into Ang-(1-7), which exerts its effects through its receptor (Mas). The opposing actions of the two receptors are listed above.

**Figure 2:**
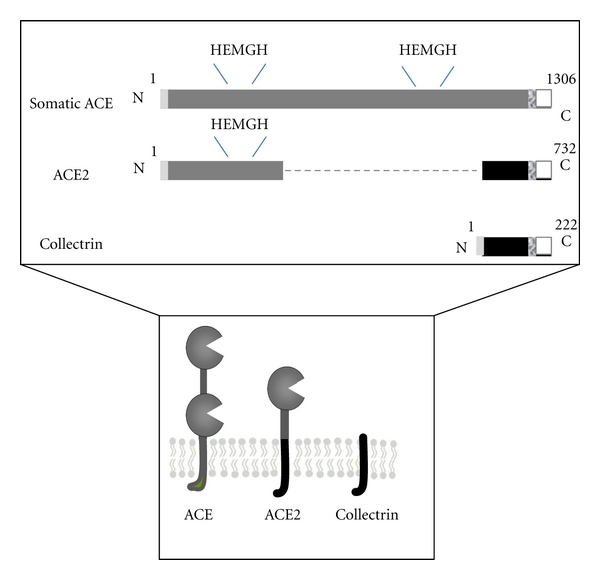
The domain structure and membrane topology of somatic ACE, ACE2, and collectrin. Each protein is a type I integral-membrane protein with an N-terminal ectodomain, a transmembrane region, and a short C-terminal cytoplasmic tail. Residue numbers are indicated. Both ACE and ACE2 contain zinc-binding motifs (HEMGH), which form the active sites of the enzyme: somatic ACE has two active sites whereas ACE2 only has one. Collectrin contains no catalytic residues. ACE2 is homologous to the N-terminal ectodomain of ACE but has no homology with its C-terminal cytoplasmic domain. Instead, it shares a number of residues with the intracellular domain of collectrin. Signal peptide in light grey; transmembrane domain in textured grey.

ACE inhibitors have been the first line of treatment against hypertension for decades, and their success has served to place ACE and its biologically active product, angiotensin II (Ang II), as central regulators of the renin-angiotensin system (RAS). Ang II is produced by ACE through hydrolysis of its precursor Ang I. Ang II is the major vasoactive peptide in the RAS, acting as a potent vasoconstrictor through its receptor AT1R (Figure 1). Hence, inhibition of the production of Ang II and more recently its receptor-induced signalling, through the use of AT1R blockers, have been highly successful treatments in hypertension. Consequently there was immediate commercial interest in ACE2, as another likely therapeutic target, when it was discovered as an active homologue of ACE. However, as the initial publications observed to their surprise, despite high similarity to ACE (Figure 2), ACE2 did not convert Ang I to Ang II nor was it inhibited by ACE inhibitors [1, 2]. A major difference in substrate specificity was immediately noticed, namely, that ACE2 acted as a carboxypeptidase removing a single amino acid from the C-terminus of susceptible substrates whereas ACE acts as a carboxy-dipeptidase (more correctly, peptidyl-dipeptidase), removing a C-terminal dipeptide. ACE2 does hydrolyse the decapeptide Ang I, albeit relatively poorly, but converts it to Ang-(1-9) rather than Ang II (Ang-(1-8)). It was initially hypothesised that ACE2 counterbalanced the actions of ACE as Ang-(1-9) is also metabolised by ACE and therefore competes with Ang I for its active site, thus providing a novel regulatory arm to the RAS (Figure 1). Studies revealed that ACE2 hydrolyses a number of substrates [3] and preferentially cleaves terminal amino acids from peptides ending in Pro-X, where X is a hydrophobic amino acid [4]. The hydrolysis of some ACE2 substrates is chloride-dependent, as is the case for ACE, and the structural basis for this selectivity has been proposed [5]. Of the biologically active peptides that ACE2 cleaves, the most relevant are apelin-13 [6] and Ang II [3]. In order to further understand the biological relevance of ACE2 an inhibitor was developed based on the C-terminal dipeptide (His-Leu) of Ang I. This allowed development of the potent and specific inhibitor, MLN-4760 [4], which has been used in numerous studies of ACE2 action *in vivo* and *in vitro*, although the compound is not currently commercially available. 

The elucidation of the structure of ACE2, and subsequent comparative modelling studies, explained its distinct specificity by revealing subtle differences in the active sites of ACE and ACE2 [7–10]. A single amino acid substitution in ACE2 sterically hinders the entrance of the penultimate substrate amino acid into the active site, thereby eliminating the ACE-like peptidyl-dipeptidase activity [7]. The substrate specificity of ACE2 was clarified when it was shown that ACE2 had a much higher catalytic efficiency for hydrolysis of Ang II (400-fold) compared with Ang I [3]. Only under conditions of elevated Ang I concentrations (such as in patients on ACE inhibitor therapy) is the conversion of Ang I to Ang-(1-9) by ACE2 (Figure 1.) likely to be of any physiological significance [11]. The revelation that the main product of the catalytic activity of ACE2 was Ang-(1-7) (Figure 1), a vasodilatory peptide, led to a complete reevaluation of its therapeutic potential. Currently strategies are aimed at upregulation of ACE2 expression and activity, technically more complex than enzyme inhibition. This does not rule out any potential application of ACE2 inhibitors which have recently been proposed as possible anti-inflammatories [12], having initially but unsuccessfully been tested as potential antiobesity drugs.

The main tissue sites of expression of ACE2 were originally identified as testis, heart, and kidney [1], where it was shown to be localised on the apical membrane of polarised cells whereas ACE is equally distributed between apical and basolateral membranes [13]. The molecular basis for this differential localization has not been addressed but presumably relates to determinants in the C-terminal cytoplasmic tails of the two enzymes which are quite distinct in sequence. The tissue distribution of ACE2 has now been catalogued more widely, for example, in liver, intestine, and lung [14, 15]. More recently ACE2 has been localized in the brain [16], where it appears to act as a central regulator of cardiovascular function [17–20]. ACE2 is a type 1 transmembrane protein (N-terminus outside, C-terminus intracellular), predominantly localised on endothelial cells where its catalytic site, like that of ACE, is exposed (so-called “ectoenzyme”) to circulating vasoactive peptides [13]. The activity of ACE2 can therefore be modulated via its expression on the cell surface, through its expression levels and also through its cleavage from the cell membrane. This cleavage or shedding releases the catalytically active ectodomain and when stimulated, for example, by phorbol esters, is mediated by a disintegrin and metalloprotease (ADAM 17) [21]. ACE also undergoes constitutive and regulated shedding from the cell surface into plasma although the enzymes responsible in this case have not been identified.

## 3. ACE2 and Cardiovascular Function

The success of ACE inhibitors has shown that Ang II is a key mediator of hypertension, and, hence, by metabolising Ang II into Ang-(1-7), ACE2 is crucial in the modulation of blood pressure. The role of ACE2 in hypertension has been clarified by its overexpression *in vivo*, reducing blood pressure in hypertensive models [22–24] but not in normotensive animals [25]. This reduction in blood pressure may be the result of increased sensitivity of the baroreflex, which has been seen upon ACE2 delivery in hypertensive models [26], and a reduction in neuronally induced hypertension has been observed in transgenic mice [19]. Central blood pressure regulation is controlled in part by the actions of Ang II on the AT1R. Ang II acts through the AT1R to desensitise the baroreflex, stimulate water uptake, and increase vasopressin release and sympathetic activation, ultimately leading to increased blood pressure [27]. The actions of Ang II are in part modulated by the increase in baroreflex sensitivity mediated by Ang-(1-7) [28, 29]. Comparison of hypertensive models to normotensive rodents has revealed decreased ACE2 protein expression by up to 40%, in the brain, of the hypertensive models [20, 22]. Moreover, overexpression of ACE2 in the brain attenuates hypertension, via an increase in nitric oxide production [19] and improved baroreflex [20]. Accordingly, injection of the ACE2 inhibitor MLN-4760 into the brain of rodents attenuates the baroreflex [18]. Site-specific overexpression of ACE2 at a locus controlling sympathetic nerve activity reduces the overall hypertensive state of rats [22]. For a review of the roles of ACE2 in central blood pressure regulation, see [30].

Soon after its discovery, gene deletion studies established ACE2 not only as a modulator of blood pressure but also as an essential regulator of cardiovascular function [31]. The progressive cardiac dysfunction observed in the first ACE2 mouse knockout model resembled that of tissue subjected to long-term hypoxia of the type that occurs after coronary artery disease or bypass surgery in humans [32]. As a result of these observations ACE2 was immediately proposed as a cardioprotective protein. This hypothesis was strengthened by the observation that ACE2-null phenotypes were reversed by concurrent knockout of the ACE gene. This evidence appeared to demonstrate unequivocally that the primary role of ACE2 was to counterbalance that of ACE [31]. The initial hypothesis that ACE2 plays a critical role in cardiac function primarily by counterbalancing the effects of ACE was not, however, entirely supported by subsequent gene deletion models. The discrepancies between the initial study and the phenotypes are described elsewhere, which saw no obvious functional or morphological changes [33, 34], were initially proposed to be due to differing genetic backgrounds in their models. This potential mechanism was investigated by backcrossing the hybrid model used in both studies with an initial parental line; however, both backcrossed models showed no cardiac changes [34]. Interestingly subsequent studies using the original ACE2-deficient mice described by Crackower et al. also showed no overt cardiac changes, suggesting that the phenotype is lost over time [35, 36]. Despite seeing no overt phenotypic change in deletion models, subsequent groups have shown a reduced ability to respond to injury in ACE2-null mice. Together these studies suggest that, rather than being a key mediator of cardiac phenotype, ACE2 is essential in modulating responses to injury [33, 37]. 

In fact ACE2 deletion models have a significantly higher mortality rate after myocardial infarction (MI) than wild-type mice, associated with adverse ventricular remodelling and worsening ventricular function following MI [38]. An increase in matrix metalloproteinase2 (MMP2) and MMP9 activation, free radical production, and upregulation of proinflammatory cytokines, in the hearts of ACE2-knockout mice, were postulated to mediate the adverse remodelling after MI. These events, and the adverse remodelling they cause, were reversed upon administration of an AT1R blocker, and therefore the pathology of ACE2 deletion in states of injury can be attributed, for the most part, to increases in the local levels of Ang II [38]. 

The ability of ACE2 to improve responses to injury is not only the result of clearing Ang II, thereby limiting its pathological potential, but also by producing Ang-(1-7). The conversion of Ang II to Ang-(1-7) by ACE2 is not the only physiological route to Ang-(1-7) production. For example, the zinc metallopeptidase neprilysin (NEP) can convert Ang I to Ang-(1-7) efficiently [39], and both ACE and NEP can convert Ang-(1-9) to Ang-(1-7). The relative importance of these various enzymes to Ang-(1-7) production will vary dependent on their relative expression levels in different tissues (e.g., kidney versus heart versus brain) and on physiological status. Like Ang II the actions of Ang-(1-7) extend beyond vasopressor control. Infusion of Ang-(1-7) reduces interstitial fibrosis in Ang II-independent [40] and Ang II-dependent hypertension [41]. Interestingly in both studies there was no effect on the blood pressure of hypertensive animals when infused with chronic levels of Ang-(1-7). There was, however, a discrepancy in the effects of Ang-(1-7) on cardiac hypertrophy between the two studies. Ang-(1-7) had no effect on the salt-induced hypertrophy in Ang II-independent hypertension but it significantly reduced myocyte hypertrophy in Ang II-induced hypertension. Cardiac-specific overexpression of Ang-(1-7) was observed to reduce the hypertrophic response to Ang II concurrently with a reduction in hypertrophic markers, atrial natriuretic peptide and brain natriuretic peptide, transcript levels and activation of hypertrophic signalling pathways, c-src and p38 MAPK [42]. Ang-(1-7) inhibits myocyte cell growth *in vitro* through the actions of the MAS receptor [43] and accordingly prevents ventricular hypertrophy *in vivo*, when stimulated by myocardial infarction (MI) [44]. The reduction in myocyte diameter and ventricular weight of mice virally expressing Ang-(1-7) was associated with a decrease in proinflammatory cytokines (TNF*α* and IL-6) compared to control. It is worth noting that Ang-(1-7) overexpression slightly reduced exogenous ACE mRNA levels and ablated the approximate twofold increase in expression resulting from MI, whilst increasing ACE2 expression levels in response to MI [44].

ACE2 levels have consistently been shown to alter in cardiovascular disease states. In light of the counterbalancing hypothesis it could be presumed that, since ACE is consistently reported to increase in damaged cardiac tissue [45–47], ACE2 levels would also increase as a homeostatic response to offset the rise in Ang II concentration. This hypothetical upregulation is supported by evidence from human nonischaemic cardiomyopathy, which has consistently shown increased ACE2 levels in the failing human heart compared to control patients [48–50]. However, in contrast, in ischaemic cardiomyopathy, there is currently conflicting evidence for the changes in expression levels of ACE2 [48, 49]. Where ACE2 upregulation has been seen in these studies, the mechanism of this damage-induced increase has been investigated using *in vivo* models of MI. ACE2 upregulation has been repeatedly shown in rat models of MI [38, 51, 52]. Discrepancies between the mRNA and protein levels seen in the infarct zone have suggested that the increase in ACE2 protein is mediated by a posttranscriptional mechanism [38]. However, time-course investigations reveal that the increases seen at eight weeks after MI were followed by a decrease in ACE2 expression in MI models compared to control after 28 weeks [52]. Although not entirely consistent these results on balance seem to indicate a compensatory role for ACE2 in conditions of myocardial injury.

Given its role in removing Ang II, ACE2 was identified as a candidate gene underlying the loci linked to hypertension [31], following its initial mapping to the X chromosome [1]. Comparison of ACE2 expression levels in the kidneys of three rat strains showed that ACE2 expression was lower in the hypertensive-prone strains and moreover that ACE2 expression decreased significantly when hypertension was initiated in salt-sensitive hypertensive rats. Decreased endogenous ACE2 expression has been noted in spontaneously hypertensive rats compared to Wistar-Kyoto [53]. The initial study did not see any genetic changes associated with the ACE2 gene in these hypertensive strains, supporting subsequent data, which have, up until now, failed to show any link between ACE2 polymorphisms and hypertension [54].

## 4. ACE2, the Kidney, and Diabetes

ACE2 is abundantly expressed in the kidneys, where its expression is inversely correlated with hypertension [55, 56]. The local RAS within the kidneys is activated by hyperglycemic conditions, which model the environment in type 2 diabetes [57]. Studies using models of type 2 diabetes have shown at early stages, prior to diabetic nephropathy developing, that ACE2 expression is reduced in the kidney, while ACE expression is elevated [58]. Similarly in models of type 1 diabetes ACE2 expression is elevated in early [59] and decreased in late stage of diabetic nephropathy [60]. Additionally, studies on human samples have shown *de novo* expression of ACE2 in the glomerular endothelium and mesangial cells of diabetic patients [61]; however, this expression was not seen in type 2 diabetic renal biopsies [57]. One study carried out by taking biopsies of twenty type 2 diabetic patients and twenty healthy donors showed decreased ACE2 and increased ACE in tubulointerstitium and glomeruli in the diabetic patients with nephropathy indicating a pathologically important balance between the two enzymes [62]. The hypothesis that kidney disease and the pathogenesis of diabetes are mediated by an upregulation of ACE and a downregulation of ACE2 was originally suggested by Mizuiri et al. [62].

As in the heart, loss of ACE2 in the kidneys is again associated with increased susceptibility to injury. ACE2-knockout mice have been shown to have enhanced susceptibility to glomerulosclerosis, coupled with increased collagen and fibronectin deposition [63]. Filtration dysfunction, evidenced by urinary albumin, was pronounced in the male mice whereas the female mice appeared to be protected. Pharmacological inhibition of ACE2, by MLN-4760, has been shown to have similar effects, increasing urinary albumin and mesangial cell expansion and vascular thickness, in both type 1 and type 2 diabetic models [58, 64]. All these studies attributed the pathology seen when ACE2 is lost to increases in levels of Ang II [58, 63]. In order to further confirm the renoprotective role of ACE2, Akita mice (a type 1 model of diabetes) were crossed with ACE2-knockout mice and kidney function observed. This model showed an increase in urinary albumin, glomerular basement membrane thickness, fibronectin, and smooth muscle *α*-actin compared to diabetic mice expressing ACE2 [35]. Surprisingly they did not see any change in Ang II in ACE2-knockouts, or in the diabetic model; despite this, they did show that use of an Ang II receptor blocker was able to attenuate some of the markers of glomerular injury and urinary albumin seen in the ACE2 knockout diabetic mice. Conversely, ACE2 deletion disrupted the benefits of ACE inhibition on diabetic nephropathy in streptozotocin-induced diabetes [65] suggesting that ACE inhibition may enhance ACE2 activity. Interestingly, in the same diabetic model, Ang-(1-7) infusion resulted in pronounced renal injury [66]. This may not be as contradictory as it first appears as they also saw a downregulation in the MAS receptor, the proposed receptor for Ang-(1-7) [67]. These current findings suggest that ACE2 may participate in a compensatory mechanism in the diabetic kidney prior to the onset of diabetic nephropathy. 

More direct involvement of ACE2 in diabetes, through its pancreatic expression, has been investigated [68]. ACE2 expression is elevated in the islets of type 2 diabetic rats, which correlates with an increase in ACE, collagen IV, and TGF-*β*1 levels [68]. ACE2-null mice have significantly increased fasting blood glucose compared to their wild-type littermates [69]. No direct role for ACE2 in the pancreas has yet been identified; in contrast its homologue collectrin is heavily implicated in insulin exocytosis. When discovered, collectrin excited interest due to its high homology to the cytoplasmic tail of ACE2 [70]. SiRNA knockdown of collectrin results in a reduction of insulin exocytosis in insulin-secreting INS-1 cells [71]. *In vivo*, overexpression of collectrin led to significant increases in insulin secretion [71]. Collectrin was implicated in the insulin secretory pathway through an association between collectrin and snapin, part of the SNARE complex [70–72]. However, collectrin-knockout mice revealed no difference in insulin secretion from wild-type, only a decrease in insulin sensitivity [73]. 

ACE2 is not only homologous to ACE but is a chimaera of ACE, with which it has close homology in the catalytic domains of the N-terminus, and of collectrin, which closely resembles the transmembrane and intracellular C-terminal domains of ACE2 (Figure 2). Collectrin was first identified as an unknown protein upregulated in a model of partial nephrectomy, its function remaining elusive for four years until crystals of tyrosine and phenylalanine were detected in the urine of collectrin-null mice [74]. Further investigation revealed that the levels of the neutral amino acid transporter, B^0^AT1, which reached the plasma membrane were significantly decreased in collectrin-null mice [75]. This suggested that collectrin may act as a molecular chaperone for B^0^AT1 in the kidney, implicating ACE2 in a similar role, because of their close homology. An elegant set of studies subsequently revealed that ACE2 did in fact act as the molecular chaperone for B^0^AT1 in the small intestine, where collectrin is not expressed. This interaction was shown to underlie the pathology of the aminoaciduria seen in Hartnup disorder. Hartnup disorder is caused by a mutation on the outer edge of B^0^AT1 resulting in its failure to reach the plasma membrane [76]. It was revealed that this mutation disrupts the ACE2/B^0^AT1 complex and therefore prevents ACE2 from acting as a molecular chaperone delivering the transporter to the intestinal brush border membrane.

**Figure 3:**
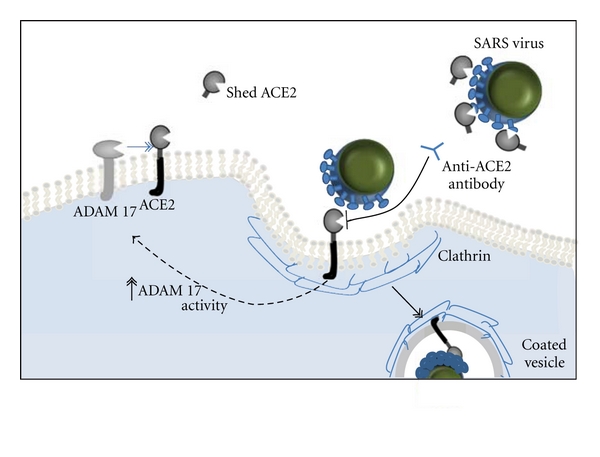
ACE2 acts as the host cell receptor for SARS-CoV, by binding to the spike protein on the viral capsid. Binding to ACE2 stimulates clathrin-dependent endocytosis of both ACE2 and the SARS-CoV, which is essential for viral infection. Binding of the spike protein to ACE2 induces ADAM 17 activity, thereby reducing the amount of ACE2 expressed on the cell surface. Treatment with soluble ACE-2 or anti-ACE-2 antibodies disrupts the interaction between virus and receptor.

Outside the cardiovascular system another noncatalytic function of ACE2 had previously been shown. In 2003 a new disease termed “severe acute respiratory syndrome (SARS)” caused by a novel coronavirus (SARS-CoV) spread quickly around the world, causing more than 800 deaths. ACE2 was identified as the receptor for SARS virus *in vitro* [77, 78] and also acts as receptor for the NL63 virus. Soon after, studies confirmed that ACE2 was essential for SARS infection *in vivo* using *ACE2*-knockout mice [79]. Concurrently it was discovered that ACE2 protects murine lungs from severe acute injury [80] and subsequently that SARS-CoV infections and the SARS spike protein itself downregulate ACE2 expression (Figure 3) [81]. 

## 5. ACE2 Regulation

### 5.1. Transcriptional Regulation of ACE2

As mentioned above there is circumstantial but not entirely consistent evidence in the literature that ACE and ACE2 are coregulated. In human hypertensive patients, ACE2 levels are lower in both kidney and heart compared to normotensive volunteers [82]. A growing body of *in vitro* evidence suggests that this decrease is mediated at least in part by Ang II [82–84]. The proposed mechanism for this involves AT1R signalling via ERK/p38 MAP [82] and/or by elevated ERK1/2 and JNK phosphorylation [85]. Furthermore, administration of an AT1R blocker has been shown to result in an increase in ACE2 levels [84, 86]. As such there is linked regulation of both ACE2 and ACE, as the catalytic product of ACE, Ang II, regulates ACE2. However, the role Ang II plays in regulating ACE2 is not yet fully elucidated; despite decreasing ACE2 expression in response to Ang II in most models there is evidence of Ang II-mediated increases in ACE2 in hepatic stellate cells [87].

Relatively little is known about the detailed transcriptional regulation of ACE2. Although angiotensin peptides, as well as other peptide and steroid hormones, appear to modulate its expression, few studies have been done on other factors that may control its regulation, such as hormones and oxygen levels. For example, although hypoxia decreases the transcription of ACE2, further investigation has revealed that hypoxia-induced HIF-1*α* increases ACE expression which, in turn, leads to an increased concentration of Ang II. It is this Ang II that then mediates a decrease in ACE2 [88]. Ang-(1-7) has also been shown to affect ACE2 expression: cardiac and renal ACE2 were decreased in both hypertensive and normotensive rat models in response to Ang-(1-7) infusion although no effects on blood pressure were demonstrated and no mechanism of action was proposed [89].

Administration of aldosterone or endothelin-1 to rat myocytes has also been shown to downregulate ACE2 mRNA levels [90]. Micromolar concentrations of aldosterone were shown to decrease ACE2 mRNA expression significantly in the myocytes of hormone-infused rats although, in contrast to other models, no change in ACE2 mRNA levels were seen when these rats were infused with Ang II [83]. When treated with endothelin-1, myocytes isolated from neonatal rats decrease ACE2 expression via ERK1/ERK2 signalling, a decrease that was blocked by cotreatment with Ang-(1-7) [90]. The effect of oestrogen on ACE2 expression has recently also been explored in light of clinical evidence which has established that hormone replacement therapy is protective against cardiovascular disease. Treating rats with oestrogen was shown to reduce cardiac remodelling and interstitial fibrosis [91]. Previous *in vitro* studies had shown that oestradiol (E2) treatment was protective against Ang II-induced fibroblast proliferation [92]. The beneficial effects of oestrogen were coupled with a dose-dependent increase in ACE2 but no significant change in blood pressure was seen and no protective mechanism proposed [91].

Components of the RAS are also expressed in adipose tissue [93]. A high-fat diet has been shown to increase ACE2 mRNA levels in mouse adipocytes both *in vivo* and *in vitro* [94] although these changes were not evident in the adipose tissues of mice with heart failure. Tissue culture models of adipose differentiation have shown that the increase in ACE2 mRNA over time was accompanied by an increase in ADAM 17 and no increase in ACE2 activity [94]. Thus the activity levels of ACE2 remain constant via coregulation with ADAM 17, which cleaves ACE2 at the cell membrane. An increase in ACE2 expression has been reported in adipose tissue when rats were fed a high-sucrose diet, although in preliminary form only [95].

The effects of all-trans-retinoic acid have been investigated on ACE2 expression revealing an increase in ACE2 mRNA levels and reportedly in protein [53]. A decrease in blood pressure in the treated rats was also seen, which was attributed to the increased ACE2 levels.

### 5.2. Posttranslational Regulation of ACE2

ACE2 expression is not only subject to posttranslational modifications, such as glycosylation and phosphorylation, but also subject to posttranslational regulation, when released from the cell membrane by shedding through the action of ADAM 17 as described above [21, 96, 97]. Cleavage of ACE2 occurs at the juxtamembrane region. Short peptide mimics around the likely cleavage site region are hydrolysed by recombinant ADAM 17 at an Arg-Ser bond (corresponding to Arg^708^ and Ser^709^ in ACE2), in a sequence-dependent manner [93, 94]. However, mutation of these critical cleavage residues in a cell-based system failed to inhibit shedding suggesting that the specificity of ADAM 17 is topographically determined, rather than sequence dependent [97, 98]. The function (if any) of the catalytically active soluble, shed form is unknown, although for some other proteins, for example, the amyloid precursor protein and acetylcholinesterase, the released protein acts as a ligand for stimulating cell-cell interactions. The retention of ACE2 on the cell membrane is regulated by calmodulin binding [99]. Inhibition of calmodulin binding increases the cellular release of ACE2. Elevated levels of shed ACE2 have been associated with increased myocardial dysfunction [100]. The catalytic activity of any shed ACE2 may be masked by the presence of an endogenous inhibitor of ACE2 in the plasma, which currently remains uncharacterised [101].

As previously mentioned SARS virus downregulates cellular expression of ACE2 [102]. Binding of the SARS spike protein induced ADAM-17-dependent shedding of ACE2 N-terminal domain [103] (Figure 3). This shedding has been reported by different groups to be both essential for viral replication [104] and unnecessary [97]. The SARS virus undergoes clathrin-dependent endocytosis upon receptor binding; this process internalises both the SARS virus and its receptor further clearing ACE2 from the cell membrane (Figure 3) and hence allowing enhanced (and damaging) reactivity towards circulating Ang II. In contrast to ectodomain shedding, the cytoplasmic domain of ACE2 appears to play no role in the regulation of internalisation [105]. 

## 6. Unanswered Questions

Currently the most pertinent of all questions about ACE2 is whether it is going to be a useful therapeutic target, and so far all data suggest that increased expression would be beneficial in a number of diseases. Until now an increase in the level of ACE2 has been achieved by viral delivery [106], application of allosteric activators of ACE2 catalysis [107], or administration of human recombinant ACE2 [108]. Aside from its effects on hypertension [22–25], viral overexpression of ACE2 has shown reduced collagen production in cultured fibroblasts [106] as well as inhibition of Ang-II-induced fibrosis and hypertrophy *in vivo* [109], stabilisation of atherosclerotic plaques [110], and renoprotection [24]. Viral delivery of ACE2 after induction of myocardial infarction is protective, reducing the adverse cardiac remodelling and fibrosis [111, 112]. Similar antifibrotic effects have been seen with an ACE2 activator [113, 114] along with attenuation of Ang-II-induced thrombus in hypertensive rats [115] and a modest reduction in the blood pressure of spontaneously hypertensive rats [107]. Over the past 18 months a number of studies have been carried out examining the effect of recombinant human ACE2 on a range of disease conditions. To date, administration of ACE2 to mouse models of Ang-II-induced diseases has been shown to reverse the pathological effects of Ang II in diabetic nephropathy [116], heart disease [117], renal oxidative stress [118] as well as reversal of Ang-II-induced hypertension [108]. Interestingly, infusion of ACE2 does not appear to have any effect on nondisease states or on the basal level of Ang II in wild-type mice or ACE2-knockout mice, making it a potentially valuable therapeutic. 

More fundamental questions about the cellular biology of ACE2 remain. As is evident from this paper, questions need answering about underlying mechanisms of ACE2 regulation, which could help clarify our understanding of the RAS as a whole, for example, are there antagonistic transcription factors regulating ACE and ACE2? Are components of the RAS co-ordinately regulated and by which signalling pathways? Do microRNAs regulate the RAS coordinately? Do cytokines modify the expression of ACE2? What posttranslational changes apart from shedding regulate ACE2 including phosphorylation and ubiquitination?

Some analogies are provided by the regulation of ACE. ACE is known to signal through phosphorylation of its cytoplasmic tail modulating its own retention on the cell membrane [119, 120] and also to mediate transmembrane signalling, increasing its own transcription in response to ACE inhibitors [121, 122]. Exogenous ACE has been shown to have transcriptional effects independent of its catalytic activity when VSMC and endothelial cells are treated with ACE [123, 124]. Does the cytoplasmic tail of ACE2, despite sharing no homology with ACE, have similar signalling properties? Both ACE and ACE2 are shed from the membrane to release their ectodomains. The fate of these ectodomains is unknown and it may be a mechanism of rapid clearance, or perhaps these ectodomains are endocytosed and trafficked to the nucleus, where they elicit transcriptional changes, as has been shown with exogenous ACE [124]. For that matter the destiny of the retained cytoplasmic sections of these proteins is not known. The intracellular domains of other shed proteins, such as APP and Notch, are released by *γ*-secretase cleavage, after initial ADAM ectodomain cleavage, a process referred to as intramembrane proteolysis [125]. The intracellular domains of both APP and Notch travel to the nucleus, stabilised by chaperones, where they elicit transcriptional changes [126]. Perhaps a similar fate awaits the ACE intracellular domain, if generated.

Novel roles for ACE2 may yet remain to be discovered and a new twist to the ACE2 story has emerged with the discovery of autoantibodies targeting ACE2 in the sera of patients with connective tissue diseases [127]. This, coupled with results showing that administration of an ACE2 inhibitor improved the pathology of inflammatory bowel disease [12], suggests that inhibition of ACE2 may be beneficial in inflammatory diseases such as arthritis. 

## 7. Summary

The diversity of the roles of ACE2 continues to surprise those in the field. Given the apparent success of recombinant human ACE2 in animal models, ACE2 could be a valuable therapeutic. However, here it is worth noting that so far the only disease model recombinant ACE2 has shown success in is type 1 diabetes. All other studies have, as yet, only shown that ACE2 reverses the effects of exogenous Ang II infusion. Until work is done on hypertensive-prone models, as with viral delivery and ACE2 activator-mediated induction, it is hard to judge the clinical relevance of this treatment. Although no immune response to recombinant ACE2 or the viral delivery systems has been reported upon infusion into rodents, this is always a concern with such strategies. Given these caveats perhaps a priority focus for future research should be upregulation of endogenous ACE2 gene expression or catalytic activity. As highlighted in this paper, more work is required to determine how the diverse roles of ACE2 interlink in order to allow chronic modulation of ACE2 levels to proceed with confidence.
